# Neurologic Music Therapy Improves Participation in Children With Severe Cerebral Palsy

**DOI:** 10.3389/fneur.2022.795533

**Published:** 2022-03-09

**Authors:** Clara Susana Santonja-Medina, Eugenio Marrades-Caballero, Fernando Santonja-Medina, Jose Manuel Sanz-Mengibar

**Affiliations:** ^1^Professional Music Conservatory Carcaixent, Valencia, Spain; ^2^Superior Music Conservatory, Valencia, Spain; ^3^Faculty of Medicine and Sports and Musculoskeletal System Research Group (RAQUIS), University of Murcia, Murcia, Spain; ^4^Department of Traumatology, V. de la Arrixaca University Hospital, Murcia, Spain; ^5^Centre for Neuromuscular Diseases, National Hospital for Neurology and Neurosurgery, UCLH NHS Foundation Trust, London, United Kingdom

**Keywords:** neurologic music therapy (NMT), therapeutic instrumental music performance (TIMP), cerebral palsy, participation, children

## Abstract

Positive effects after neurologic music therapy (NMT) have been described regarding the motor function of children with severe cerebral palsy (CP). This study aimed to quantify improvements in participation, as well as complexity on task-related manual activities in children with severe bilateral CP. This analytic quasi-experimental study exposed 17 children with severe cerebral palsy to 13 NMT sessions to improve motor learning through therapeutic instrumental music performance (TIMP), using principally percussion musical instruments. Hoisan software video recording was used to quantify participation involved in creating music. In addition, the number of active movements performed in each NMT session was quantified. Significant improvements were found in the participation variables “visual contact,” “motor participation” and “motor participation repetitions.” Significant differences were also found in the subcategory “reaching and stroke,” “hitting with the hand” and “grasping and hitting.” The use of therapeutic of TIMP in children with severe CP improves participation during manual activities utilizing percussion instruments, therefore increasing the intensity of the psychomotor intervention.

## Introduction

Improvements in gross motor function (1, 2) and gait (3, 4) in children with moderate cerebral palsy (CP) have been described after implementing neurologic music therapy (NMT) techniques (5). More recently, these results were observed after using therapeutic instrumental music performance (TIMP) and clinical improvisation in children with a poor gross motor, manual and communication skills (6). Functionality have an important role to categorize cerebral palsy into different functional levels and these children were classified within Gross Motor Function Classification System (GMFCS-E&R=IV-V) (7), with severely limited manual ability [Manual Ability Classification System (MACS)=IV-V] according to Eliasson et al. (8), and severe impairment according to the Communication Function Classification System (CFCS) as stated by Cooley Hidecker et al. (9). The positive effects in terms of neuroplasticity following active musical training with musical instruments have been attributed to the consistent bidirectional transmission of information between the sensory and motor areas of the brain (10) and the effect of hearing and visual feedback (11). Changes in the internal mechanisms underlying motor function can explain functional improvements in CP due to rehabilitative intervention through musical training with instruments (12, 13).

The rhythm acts as a force that links motor and cognitive aspects through neurophysiological processes such as priming (5, 14) and entrainment (15–17). Musical listening and active movement could facilitate the priming of the neural processes of entrainment. This combination has shown positive results during NMT techniques, including Rhythmic Auditory Stimulaiton (18). The same entrainment rhythmic properties are included in TIMP, as well as the regulation of the spatio-temporal aspects of the motor control. TIMP facilitates a higher temporal processing because the patients move synchronically with the sensorial rhythm and take part on production of the music pattern (5).

The active participation and motivation of children with severe motor and attentional difficulties is not easy during rehabilitative tasks. Repetition and increasing task difficulty are limited, although these are important factors for a successful intervention (19). Patients with severe CP and very limited functional capacity (GMFCS, MACS and CFCS levels IV and V) often lack the motivation to repeat active voluntary movements (20). This is aggravated by limited social interaction due to communication difficulties, even in familiar environments (9). Attention span is noticeably reduced, as well as the development of their need to participate during the activities of daily living. This vicious circle worsens the problem and exacerbates musculoskeletal deformities (20).

Active participation is highly enhanced in neurorehabilitation, integrating perceptual skills, memory, emotions and central nervous system regulation systems (21), but requires a certain degree of motor control. Difficulties in the coordination of voluntary movements could be due to cognitive aspects, or due to a lack of timing adaptation during muscle activation to initiate, maintain and stop a movement (22). The structured succession of musical sounds can facilitate the coordination and synchronization of movements and improve emotional processes (5, 23). TIMP techniques in rehabilitation can develop self-expression mechanisms (24), target regions where volition and motivation originate (25), and improve movement planning and coordination (5).

TIMP designs physical exercises aiming to play with musical instruments that emulate other functional movement. Percussion instrument are easier to access by children with severe CP, facilitating participation in musical and creative activities, improvised or predefined, while performing functional movements (5). In this Neurologic Music Therapy technique, musical instruments such as drums or keyboard are rather placed in strategic locations relative to the patient's body to train range of motion, functional movements and limb coordination (26).

Feasibility study protocols and several lab-based studies with stroke patients have produced statistically significant gains in upper limb function when using TIMP (27). These studies included 30 min to 1 h sessions using acoustic instruments and MIDI drums (28, 29) and different TIMP protocols were carried out (30–33). Other studies in more severe patient with stroke describe how musical experiences motivated patients to be engaged in the activity and to repeat the targeted functional movements (29, 34). The use of TIMP in neurological rehabilitation could contribute to the improvements on functional motor performance of children with severe CP (6). Increasing participation and task complexity have to be considered during this intervention, but these factors are severely limited in these patients (19).

A systematic literature review by Weller and Baker (35) about the role of music therapy in physical rehabilitation encouraged the quantification of interrelated variables during the therapeutic process.

Significant improvements in function and mobility in children with severe CP after therapeutic intervention with percussion instruments justify further research on other observed psychomotor aspects that need to be quantified. Matney (36) said “there currently exists a need to better understand the therapeutic applications and therapeutic function of percussion instruments in therapy,” This is explained in children with severe CP because musical instruments can be used as facilitator of psychomotor task-oriented motor learning (6). Percussion instruments facilitate physicality because a wide range of movements is required to play the instruments. In addition, they help to improve non-verbal communication and social skills (37). Training exercises involve a strong rhythmic component. Music is used to provide rhythmic cues to facilitate auditory-motor entrainment (26).

Our hypothesis is that NMT enhances participation during task-related manual activities and facilitates increasing complexity. This study aimed to quantify participation and complexity improvement during task-related manual activities using TIMP in children with severe bilateral CP.

## Materials and Methods

### Study Design and Participants

This analytic quasi-experimental study used both pre-intervention and post-intervention measurements without a randomly selected control group. This analytic design study NMT as etiological factor to improve the clinical picture of the subjects. Twenty-four children were assessed before and after a period of 4 months when they received their usual therapeutic input. After this unaltered period, NMT intervention was added without modifications on the previous program. This quantitative design not only solved the ethical limitations of considering a control group without the requested intervention, but also turns every subject in his/her own control, with the same personal factors and therapeutically conditions. Children were recruited from “La Cruz Roja Cerebral Palsy Center”, Valencia (Spain). Inclusion criteria were: aged between 4 and 18 years old, a diagnosis of bilateral CP, severe degree of gross motor function (GMFCS-E&R), severely impaired manual ability (MACS), severe to moderate degree of communication function (CFCS), hearing preservation, interest in music and no risk of self-harming. In addition, participant's parents or guardians gave informed consent for participation in the study and video recording.

**Figure 1:**
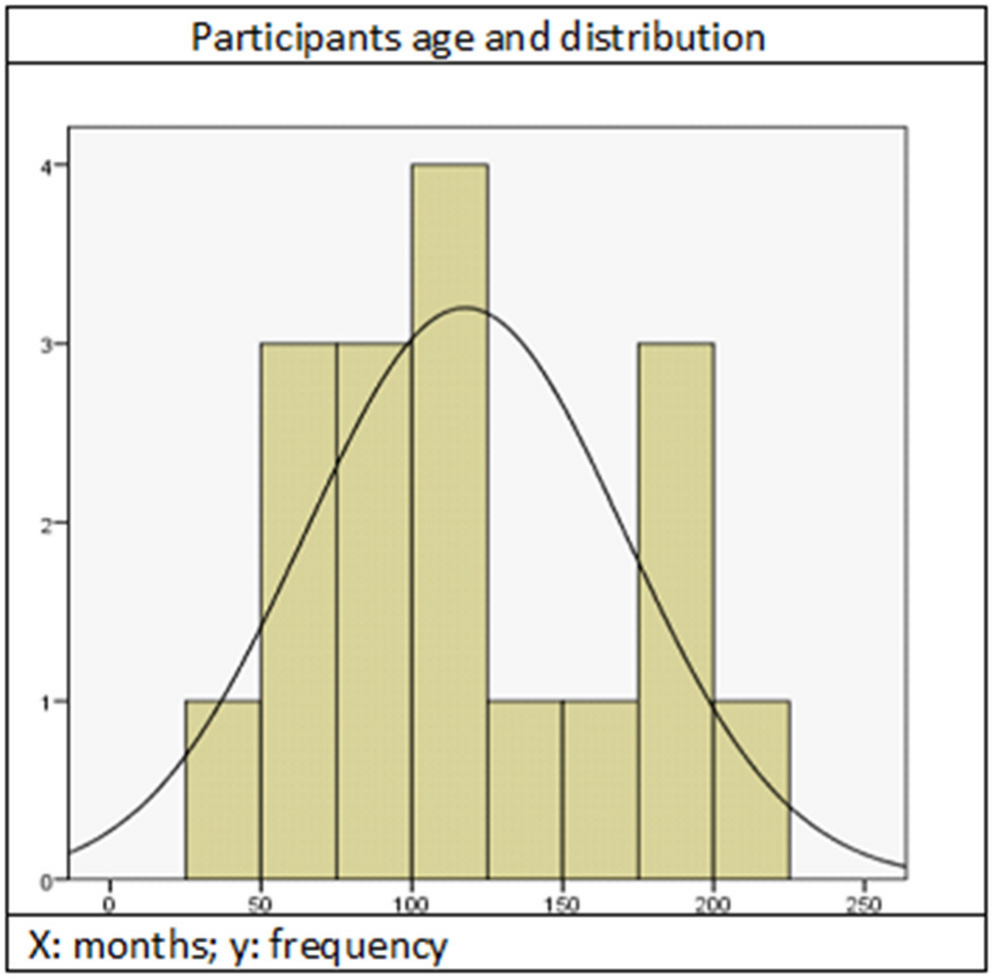
Participants age and distribution. X, months; Y, frequency.

Exclusion criteria were: severe behavioral problems, poor attention due to secondary effects of medication and non-attendance at more than 20% of the sessions. Only 17 patients met the inclusion criteria (12 males and five females), but two had to be excluded due to visual impairments. Sample features are summarized in Table 1, Figure 1.

**Table 1:** Sample distribution.

	**GMFCS**		**MACS**		**CFCS**		
**Level**	**IV**	**V**	**IV**	**V**	**III**	**IV**	**V**
**Male**	2	10	3	9	2	4	6
**Female**		5	3	2			5

### Pre-intervention

A preliminary study of the variables to quantify was performed 4 months before the first NMT intervention. This previous data collection allowed us to understand how the studied parameters changed when NMT was still non-implemented. During these 4 months, the children received only their usual therapeutic inputs and each subject would later on be comparable to their own control data. The measurements of the first pre-NMT intervention session were compared with the initial assessment 4 months before. Cohen's d was obtained for all the variables using the standardized mean difference (Cohen's d) and according to Hopkins et al. (38); the effect of this value was considered trivial (*d* values < 0.2). Visual contact difficulties were observed as the children could not hold a stare for more than 1 or 2 s toward the instruments, therapists or other sources of sound. This limited interaction and intentional participation was better when they could focus for more than 3 s. Therefore, a threshold of 3 s was established to measure significant differences in sustained visual contact and the duration of intentional motor participation.

Gross motor, manual and communication classifications (GMFCS, MACS and CFCS) were also obtained at this initial assessment. Musical habits and preferences of each child were also inquired to parents or carers.

### Intervention

Forty-minute customized one-to-one sessions were performed once a week for 4 months. A total of thirteen NMT sessions were carried out simultaneously by a team of two music therapists who facilitated the use of musical instruments for upper limb tasks to improve motor learning. Several repetitions of these activities were performed, using auditory stimuli. The exercises with musical instruments were specifically designed to enhance participation and encourage movement, as well as increase the level of difficulty progressively. The music was always live and customized according to each patient's needs and preferences. The therapist's voice, small percussion instruments, Spanish guitar, keyboard (Casio Tone Bank-6) and digital table drums (e-drum 150), were used. Each session included TIMP activities that facilitated specific tasks with the upper limbs.

### Procedure

Activities were performed in the prone position on a mat and sitting on their own adapted chairs. Resting periods (1 to 3 min) were adapted according to each child needs, as well as the task-specific activities and musical instruments with the goal of maximize the progression in every session. Sessions were video-recorded and analyzed afterwards to prepare the following intervention, define the functional goals, understand which movements where difficult for further practice and sequencing more complex functional tasks. Specific musical activities were then design according to these parameters, as well as width, weight, location-space and timbre adaptations. Due to attention span, concentration and motivation difficulties, warm up activities were included at the beginning of each session with the goal of facilitate TIMP implementation.

### Intervention Protocol

#### Warm Up Activities

The children were approached with a “welcome song” performed by the music therapist with a binary rhythm and emphasizing the pulse to engage them emotionally. The sounds created by the rhythmic beat were easily recognizable for the patients and facilitate priming and voluntary movements. Task-specific activities and more suitable instruments were tried out for each child in order to progress more efficiently. Children created rhythmic motifs with spontaneous basic combinations using their favorite instruments and their own strategies of movement. The music therapists encouraged them with their voices, prosodic expressions, the guitar and percussion instruments. This provided a live musical structure and aesthetic function to the self-generated rhythmic motifs. Instruments were previously adapted to individual preferences, in order to facilitate their musical interaction and integrate them as essential component of the new created musical piece. These activities tried to achieve motivational support and to open new channels of expression as well (24). Interventions were defined with the goal of increasing the participation of the children. The structure of these activities aimed to facilitate visual contact and up-righting orientation of the head and trunk toward the instruments before the movement of the upper limbs. The music therapist initiated placing the instruments at the level of the eye and then moving them to different heights. The visual-hearing interaction included also different timbres and intensities. Children were also encouraged to press the keyboard or hit the drum to facilitate the vertical movement of the upper limbs. Therapists assisted the posture of the children with their hands if needed, and this support was reduced when the motivation to hold their trunk increased.

#### TIMP Intervention

TIMP stimuli may be created by a specific compositional or improvisational process that the therapist uses to accommodate the specific needs of patients (5). Training exercises involve a strong rhythmic component. Music provide rhythmic cues to facilitate auditory-motor entrainment. Cueing frequency is initially matched to the comfort level and gradually decreased/increased depending on the therapy goal (26).

Then, specific tasks for unilateral reaching-stroking, hitting, grasping and holding and intermediate object and hitting a musical instrument were carried out. To that end, the therapists created live musical pieces with rhythmic patterns. Accents, upbeats and dynamic adjustments were introduced to guide the start and the end of the movements. Music therapists matched their voice and music with the beat, with the dynamic intensity, and complexity level according to each child. A rhythmic phrasing was chosen and adapted to each of them to integrate and synchronized the frequency of their beats. Subsequently, leader-follower roles were interexchanged through musical games for holding or alternating the rhythmic patterns. These activities facilitated our aim of improving “sustained visual contact” (5).

Encouraging words and facial expressions were used by the therapists while placing the musical instruments and sticks according to the observed needs. They adapted the height, the position as well as the shape and width of the sticks. This adaptation aimed at progressive accessibility to the percussion instruments and increasing difficulty levels during both improvisation and structured therapy.

Table 2 describes more specifically the intervention protocol according to the session progression.

**Table 2:** Intervention protocol according to session progression.

**Intervention protocol 1: enhancing uprighted position**	
**Session number 1**	**Warm Up**
**Tasks' Goals**	**Holding head and trunk control against gravity for longer periods Holding visual contact for longer and orientation from mid line as much as possible**
**Musical intervention**	**- Auditive and visual stimuli at different heights. Musical Instruments: Music Therapist voice, triangles, guitar, keyboards, bells and cymbals** **- Predictable and unpredictable emphasis while keeping a regular pulse** - **Random participations of the patient generally requiring holding upright while pressing on the instrument**
**Therapist assistance**	**- The trunk of the patient can be assisted from the shoulders by the Music Therapist, avoiding forward or lateral tilt.** **- The Music Therapist support one arm during bilateral activities, while the patient focus on the other arm** **- Support is reduced when the patients feel more confident during the task**
**Adaptations**	**- Instruments are presented on the midline or laterally for the patient to touch or press**
**Intervention protocol 2: reaching with the upper limb**	
**Session number 2-3-4**	**TIMP**
**Tasks' Goals**	**Unilateral and selective orientation of the arm toward the musical instrument**
**Musical intervention**	**- Music Therapists perform melodic ascendant voices with harmonic foundations progressing up and down from 5^th^ (guitar) to guide the movement** **- Emphasis and anacrusis to indicate when the movement start. Dynamic intensification of the stroke** **- Music Therapists play and sing at the same tempo, dynamic, texture and level of complexity develop for the patient** **- Develop a rhythmic and harmonic tone background** **- Every movement is positively feedbacked with their favorite keyboard, drum or cymbal sound**.
**Therapist assistance**	**- Rhythmic background with bongos** **- Activity mirroring, facial expression and prosodic expressions** **- Positive feedback with their favorite song after trying- effort**.
**Adaptations**	**- Instruments are presented at different locations, height** **- Favorite sounds and timbres**
**Intervention protocol 3: hitting with the upper limb**	
**Session number 5-6-7-8**	**TIMP**
**Tasks' Goals**	**Unilateral and selective orientation of the arm to produce sounds with the instruments**
**Musical intervention**	**- Drums or bongo are presented at different locations to required more complex arm positioning** **- Drums and tambourine are played by the Music Therapists, creating rhythmic combinations and emphasis of 2-3 beats, creating asking-answer and turn taking activities** **- Roles exchange between patient and Music Therapists**
**Therapist assistance**	**- Rhythmic pulse** **- Facilitation of the role exchange** **- Keeping motivation level** **- Support the instrument location and positioning**
**Adaptations**	**- Instruments are presented at different locations and height, but also into more challenging position and easier depending on motivation and motor possibilities**
**Intervention protocol 4: grasping and hitting with intermediate objects with the upper limb**	
**Session number 9-10-11-12**	**TIMP**
**Tasks' Goals**	**Unilateral and selective orientation of the arm to produce sounds with the instruments using and intermediate object. Playing instrument in different positions**
**Musical intervention**	**- Patient plays the drums or bongo using stick, at different locations to required more complex arm positioning** **- Patient lying in prone or supine while stroking, hitting and grasping or using the keyboard (Casio Tone bank-6). Taking turns or asking-answer activities.** **- Patient sitting and using stick to play e-Drum DD 150.** **- Music Therapist answer rhythmically to develop and generate the musical tasks**
**Therapist assistance**	**- Stick need to reach and hit the instruments farther and in more challenging positions of the upper limbs and graps (Drums, keaboard)** **- Communication feedback** **- Support motivation** **- Support to grasp the sticks accordingly**
**Adaptations**	**- Stick size-width Slope–wedge cushion to adapt lying posture.** **- Keyboard and drums position at different heights and distances** **- Keyboard sounds adapted to patient's preferences**
**Intervention protocol 5: description of new goals**	
**Session number 13**	**Activity summary and intervention closing**


### Assessment of Outcomes

Hoisan (Tool Observation Social Interaction in Natural Environments) (39) is a software application that enables de-encoding, recording, description and handling of video recordings. Two cameras were used to quantify in seconds the participation variables according to the International Classification of Functioning, Disability and Health (40). Children “participated” if they maintained a stare at the source of sound (visual orientation) or create sounds with a musical instrument for at least 3 s (motor participation). The number of participative repetitions of each subject was also quantified.

Participation was organized hierarchically according to four levels of task difficulty:

(1) Visual contact with the task for at least 3 s.
(2) Reaching for and stroking a musical instrument with either hand for at least 3 s.
(3) Hitting a musical instrument with the hand for at least 3 s.
(4) Grasping and holding an intermediate object and hitting a musical instrument with it for at least 3 s.

Reaching-stroking, hitting and grasping were considered progressively more difficult subcategories, in order to quantify the complexity of the motor participation.

Video recordings of their last session were used for the post-intervention assessment; however, to get used to the new therapist and intervention, the second session was selected for the pre-test assessment. Video recordings of the children were randomized to blind the therapist-rater to the pre- or post-intervention assessment. With the goal of attaining even higher levels of verification of these assessment, nine of the 17 (52.9%) video recordings were randomly selected and scored again after 6 months, to calculate the intra-rater reliability of the examiner.

This study was approved by the Research Ethics Committee of [institution name removed for purposes of blind review].

### Statistical Analysis

A descriptive statistical analysis was performed to obtain the frequency distribution of each variable. Average, minimum and maximum values, as well as standard deviations were calculated.

The test of normality was performed with Shapiro-Wilk for all the studied variables. Consequently, data analysis was performed with non-parametric or distribution-free tests. Wilcoxon signed rank test was used to determine the effect of NMT. Also, the effect size between the pre and post-test for all variables was calculated with the standardized mean difference (Cohen's d). The effect size was interpreted according to Hopkins et al. (38): an effect size lower than 0.2 was considered as trivial; values between 0.2 and 0.59 were considered poorly related; between 0.6 and 1.19 had a moderate effect; between 1.20 and 2.00 was strong; between 2.00 and 3.99 was very strongly related; values higher that 4.00 indicate extremely related. These authors chose “moderate” as the minimum relevant size effect to consider the results of an intervention valid.

Paired-sample *t*-tests were performed with the goal of verifying differences due to the intervention in the different categories.

The random effects model of the intraclass correlation coefficient (ICC) was used to verify the intra-rater reliability after two scorings of the same videos.

Statistical analyses were performed using SPSS, v. 25 (IBM) and only *p* < 0.05 was considered statistically significant.

## Results

Significant differences after the NMT intervention program were found in the variables “visual contact” (*p* < 0.0005), “motor participation” (*p* < 0.0005) and “repetition/number of motor participations” (*p* < 0.004). Significant differences were also found in the motor participation subcategories “reaching and stroke” (*p* < 0.0015), “hitting with the hand” (*p* < 0.023) and “grasping and hitting” (*p* < 0.017) (Table 3). The “visual contact” variable showed a strong effect size (d = 1.21), as did “participation” (d = 1.24). Moderate “repetitions/number of participations” (d = 0.86) indicated the relevant effect of increasing visual contact on participation and the number of repetitions after TIMP intervention and clinical improvisation with percussion instruments (Table 3).

**Table 3:** Quantification of the participation categories at pre and post intervention.

**Participation category**	**Average**	***n***	**SD**	**Standard error mean**	***p*-value**	**SMD (d)**
Visual contact	seconds					
Pre-test	472,53	15	489,16	126,30	0.0005^*^	
Post- test	1143,87	15	613,43	158,39		1,21
Reaching and stroke	seconds					
Pre-test	229,24	17	351,48	85,25	0.015^*^	
Post-test	428,35	17	456,57	110,73		0.49
Hitting with the hand	seconds					
Pre-test	60,53	17	82,59	20,03	0.023^*^	
Post-test	214,82	17	264,53	64,16		0.79
Grasping and hitting with an object	seconds					
Pre-test	115,53	17	240,81	58,40	0.017^*^	
Post-test	364,53	17	493,31	119,65		0.64
Variab. Total Motor Participation	seconds					
Pre-test	405,29	17	366,71	88,94	0.0005^*^	
Post-test	1007,71	17	579,87	140,64		1,24
Strokes repetitions	number					
Pre-test	81,41	17	101,88	24,71	0.156	
Post-test	121,29	17	146,11	35,44		0.32
Hits with the hand repetitions	number					
Pre-test	49,76	17	83,10	20,16	0.081	
Post-test	143,76	17	196,83	47,74		0.62
Grasps and hits repetitions	number					
Pre-test	73,12	17	157,62	38,23	0.077	
Post-test	336,18	17	655,38	158,95		0.55
Total number of Motor repetitions	number					
Pre-test	204,29	17	192,23	46,62	0.004^*^	
Post-test	601,24	17	625,02	151,59		0.86

*SD, Standard Deviation;*

* *p < 0.05; SMD (d), Standardized mean difference*.

A moderate effect size was observed in the participation subcategories: “hitting with the hand” (d = 0.78) and “grasping and hitting with an object” (d = 0.64). In the subcategory “reaching and stroke,” only a poor effect size (d = 0.49) was obtained. In the repetition category, the effects were moderate only for the subcategory “hits with the hand” (d = 0.62) and poor for both “number of strokes” (d = 0.32) and “number of grasps and hits” (d = 0.55).

No statistically significant differences were found after the NMT intervention in the following variables: “total number of strokes,” “total number of hitting with the hand” and “total number of grasping and hitting with an object;” nevertheless, an overall tendency toward statistical significance was observed.

## Discussion

Previous research hypothesized that music is employed to connect the physiological, psychological, cognitive and emotional components of physical rehabilitation (35). The results obtained in our study confirmed our hypothesis: the systematic combination of NMT techniques using musical instruments therapeutically facilitated the psychomotor aspects of manual function in our children and adolescents with severe bilateral CP. This research shows, for the first time in this group of children, the positive effects of TIMP intervention on participation in a manual task (reaching, stroking, grasping and hitting).

Significant improvements in the willingness to participate were confirmed from different aspects while playing musical instruments: duration of participation (*p* = 0.0005) and number of attempts (*p* = 0.004) to stroke, hit or gasp intermediate objects to hit the instrument. These facts and the relevant effect size showed by the standardized mean difference (Cohen's d) should make us think of NMT as a complementary treatment and as a support for manual daily living activities.

NMT significantly increased the visual contact of our subjects during manual activities; this factor is essential to succeed with any therapeutic intervention (19). Specific tests to quantify attention capacities in our sample have not been found. Bottcher (41) used the Test of Everyday Attention for Children (TEA-CH) in subjects with GMFCS I-II-III (94%); however, this scale was not applicable to our sample due to severe motor difficulties classified as GMFCS IV and V (V = 93.33%). We therefore timed intentional visual contact toward the source of the sounds to measure the attention span. Non-intentional staring or shorter than 3 s was not quantified according to our previous analysis 4 months before the intervention.

Other cognitive aspects such as memory and executive capacity seem to be enhanced by NMT because they take place during motor learning processes (42, 43). Memory was not quantified in our study, but we can assume that improvements were also achieved in this aspect after repetitive activities because anticipation and self-decision making were observed. On the other hand, motivation to complete more difficult tasks suggests the reinforcement of executive capacity. The possibility of increasing the intensity of the intervention is a basic requirement to optimize the therapeutic process (44).

Children with CP are among the populations least served by music therapy services in neurorehabilitation. More research into cognitive and communication rehabilitation are required in order to better understand the symptomatic findings in this neurological condition (45). Interrelations among rhythm, movement, cognitive and socio-emotional aspects (15) were achieved in our study. This process implies the understanding of proposed activities, which is a difficult requirement considering the severe communication difficulties of our sample. Most of our children had no verbal communication and found it difficult to communicate even in a familiar environment. The music therapistS had to develop a communication channel with them through the music. Longer participation times and increases in the number of movements indicated better understanding of the instructions regarding the musical resources.

An increase in the intensity of therapeutic practice has been recommended in neurorehabilitation (44), and this was intended with the NMT sessions; nevertheless, it was important not to reduce the level of visual contact when increasing the difficulty due to the features of our studied sample (6). Different studies (12, 31–34, 46, 47) indicate an approximate frequency of intervention sessions of 40 min, twice a week for a period of between 3 or 4 months. However, some authors (31, 32) argue that the clinical stage, the severity of the case, the duration of treatment and the dose should be considered individually to better adjust the intervention to the capacity of each individual. In our study we carry out individual sessions of 40 min, once a week, in order to mitigate the effect of fatigue that our patients present severe cerebral palsy. This frequency of treatment has shown significant improvements in carrying out activities in this type of patient (6).

Therapeutic techniques with musical instruments were mixed with resting periods during our interventions, avoiding eventual fatigue. This fact allowed children to interact for 40 min in the sessions. TIMP filled 60% of the sessions (25 min). The remaining 40% (15 min) was distributed over the warm up, rest, summary and preparation for the next session.

The cooperative work between both music therapists was key to break through the communicative possibilities. Musical, expressive and prosodic resources reflected the children's mood, behavior and attitudes in order to empathized with them. This contributed to improve their motivation, participation and emotional stability, as well as avoiding “acting out.” Improving bonding helped also with their arousal and predisposition to movement because their needs were immediately satisfied. The posterior analysis of the video recorded sessions was required to understand with more details their behavior and improve further communication, and reduced the chances of therapist “burn out.”

Each patient presented unique psychomotor features and compensated movements with specific strategies. Using this freedom to play the musical instruments (warm up) provided them with intrinsic motivation when they perceived the benefits of creativity. This also encouraged repetition of the trained movements with TIMP but avoided routine or stereotypic behavior. The motivation acquired during easier tasks was useful to move on to higher levels of complexity. In this way, two important requirements in neurorehabilitation were provided, i.e., repetition and a progressive increase in the difficulty of the activities (19).

A total of 1,937 music therapy interventions with percussion instruments were mentioned in Matney's review (36), of which only 26 were electronic. Our study included analog instruments (bongo drums for instance) that could be placed according to the patient's requirements, but also digital instruments (an e-drum 150 and Casio tone keyboard). These provided a clearer sound because sound intensity could be adjusted and provided better registration of the hits.

The ethical limitations of randomized untreated control groups or placebo interventions limit the quality of the intervention in this field. The current assessment of the cognitive aspects was not controlled, and this could be considered a limitation of the current study. Specific improvements in visual contact and manual performance are described here under the same personal factors and therapeutic conditions, but a one-to-one control without NMT intervention is not available.

The sample size was a limitation of our study (*n* = 17). Our sample size is similar or even larger than previous research using NMT techniques in children with less severe CP. Kwak (3) treated 16 subjects with movement and music, Kim et al. (4) applied rhythmic auditory stimulation (RAS) in 15 subjects and improvements in walking capacity were described in both studies. The effects of patterned sensory enhancement (PSE) were studied by Peng et al. (2) in 23 subjects who could stand independently and by Wang et al. (1) in 18 subjects who were able to transfer from sitting to standing. Chong et al. (48) used TIMP with only five subjects who could sit unassisted at the keyboard and were able to communicate verbally. Marrades-Caballero et al. (6) described improvements in the motor function of 18 children with severe CP using TIMP. Efraimidou et al. (49) described the effects of a music and movement intervention program on gait, balance and psychological parameters of 10 athletes with CP, showing the relevant difficulties of these subjects.

Increasing the number and intensity of the sessions could help to support our results showing performance on music tasks transfers into daily living activities at severe levels of the GMFCS and MACS (IV-V) and how NMT can support communication skills. Eight subjects out of 17 increased their performance level after TIMP intervention, starting from not even reaching: two children achieved stroking, another two children accomplished hitting with the hand, and four even attained grasping and hitting. Only 11 out of 540 studies using percussion instruments therapeutically were aimed at subjects with CP (36). Of them, only one included children severely affected in terms of their gross motor, manual and communication functions (6).

Further research is needed to explore the benefits of using TIMP in people with CP, including other parameters such as active range of motion. Studies should recruit subjects with other neurological conditions who may benefit from a methodical intervention with instruments for therapeutic purposes.

## Conclusion

This study provides preliminary evidence that the therapeutic use of NMT active techniques, using percussion instruments for children with severe bilateral CP, improves participation during manual activities. Psychomotor rehabilitation will benefit from these requirements because the level of difficulty, the number of repetitions and the duration of participation during task-specific motor learning can be increased.

## Data Availability Statement

The raw data supporting the conclusions of this article will be made available by the authors, without undue reservation.

## Ethics Statement

The studies involving human participants were reviewed and approved by Research Ethics Committee of the University of Murcia ID: 1186/2015. Written informed consent to participate in this study was provided by the participants' legal guardian/next of kin.

## Author Contributions

All authors performed design, intervention, data analysis, and article writing. All authors contributed to the article and approved the submitted version.

## Conflict of Interest

The authors declare that the research was conducted in the absence of any commercial or financial relationships that could be construed as a potential conflict of interest.

## Publisher's Note

All claims expressed in this article are solely those of the authors and do not necessarily represent those of their affiliated organizations, or those of the publisher, the editors and the reviewers. Any product that may be evaluated in this article, or claim that may be made by its manufacturer, is not guaranteed or endorsed by the publisher.
